# Overlap of Asthma and Chronic Obstructive Pulmonary Disease in Patients in the United States: Analysis of Prevalence, Features, and Subtypes

**DOI:** 10.2196/publichealth.9930

**Published:** 2018-08-20

**Authors:** Ralph M Turner, Michael DePietro, Bo Ding

**Affiliations:** ^1^ HealthCore, Inc Wilmington, DE United States; ^2^ Teva Pharmaceuticals Malvern, PA United States; ^3^ AstraZeneca Pharmaceuticals Gothenburg Sweden

**Keywords:** COPD, asthma, asthma-COPD overlap, ACO, claims data, medical records, diagnosis validation

## Abstract

**Background:**

Although asthma and chronic obstructive pulmonary disease (COPD) are clinically distinct diseases, they represent biologically diverse and overlapping clinical entities and it has been observed that they often co-occur. Some research and theorizing suggest there is a common comorbid condition termed asthma-chronic obstructive pulmonary disease overlap (ACO). However, the existence of ACO is controversial.

**Objective:**

The objective of this study is to describe patient characteristics and estimate prevalence, health care utilization, and costs of ACO using claims-based diagnoses confirmed with medical record information.

**Methods:**

Eligible patients were commercial US health plan enrollees; ≥40 years; had asthma, COPD, or ACO; ≥3 prescription fills for asthma/COPD medications; and ≥2 spirometry tests. Records for a random sample of 5000 patients with ACO were reviewed to validate claims-based diagnoses.

**Results:**

The estimated ACO prevalence was 6% (estimated 10,250/183,521) among 183,521 full study patients. In the claims-based cohorts, the comorbidity burden for ACO was greater versus asthma but similar to COPD cohorts. Medication utilization was higher in ACO versus asthma and COPD. Mean total health care costs were significantly higher for ACO versus asthma but similar to COPD. In confirmed diagnoses cohorts, mean total health care costs (medical plus pharmacy) were lower for ACO versus COPD but similar to asthma (US $20,035; *P*=.56). Among confirmed cases, where there was medical record evidence, smoking history was higher in ACO (300/343, 87.5%) versus asthma cohorts (100/181, 55.2%) but similar to COPD (68/84, 81%).

**Conclusions:**

ACO had more comorbidities, medication utilization, and costs than patients with asthma or COPD but differences were not seen after confirmation with medical records.

## Introduction

Obstructive lung disease is a significant public health problem. Combined, airway diseases such as asthma and chronic obstructive pulmonary disease (COPD) affect up to 15% of adults in the United States, cause more than a million hospitalizations, and over 15 million lost work days [1]. The global effects of combined asthma and COPD are even more dramatic—300 million people are affected by COPD, and up to 300 million by asthma. COPD, the third leading cause of death worldwide, is associated with an estimated 3 million deaths per year, and asthma with 200,000 deaths per year [2].

Although asthma and COPD are clinically distinct diseases, they represent biologically diverse and overlapping clinical entities. Clinicians have been studying asthma and COPD in relation to each other for more than half a century, since the formulation of the Dutch hypothesis in 1961 [3,4]. Asthma and COPD overlap [5] commands considerable attention and is discussed comprehensively in guidelines such as the Global Initiative for Asthma [6] and the Global Initiative for Lung Disease for COPD [7].

Asthma-COPD overlap (ACO; previously referred to as asthma-COPD overlap syndrome) is characterized by persistent airflow limitation consistent with COPD, together with several distinguishing features of asthma [7]. Prevalence estimates for ACO range from 5.5% to 55% [8-14] and the large discrepancy islikely attributable to differences in diagnostic criteria for asthma and COPD [5] and other factors, including age and gender [15]. Despite a growing body of literature, no standard exists to identify the syndrome and there is no consensus definition [16]. The result is a mixed picture of overlapping symptoms, patient characteristics, and comorbidities not reliably differentiated from asthma and COPD [17].

The literature suggests that, compared with asthma or COPD, ACO is associated with more rapid decline in lung function, more frequent exacerbations, increased health care resource utilization, worsening quality of life, and higher mortality rates [16,18,19]. This profile, however, relies on diversely defined populations and prevalence estimates [15,17,20] and might have dubious diagnostic utility.

The treatment responses of patients with ACO could be important for clinical decisions, suggesting potential value in additional, more precise characterization of this disease entity [5,15]. Little epidemiologic research has critically evaluated patterns of clinical diagnosis using the commonly used overlapping International Classification of Diseases, Ninth Revision (ICD-9) code patterns for asthma and COPD which can suggest possible ACO, indicating an important gap in knowledge. Such patterns of diagnosis might provide additional clues to better characterize the disease entity. A better understanding of the features of ACO might lead to improved diagnosis and treatment of this entity and improvements in public health for those patients affected by airways disease

Respiratory diseases, notably asthma and COPD, have resulted in immense clinical and economic challenges for public health [21,22]. Health services vigilantly investigate and seek to understand the epidemiological trends of respiratory diseases in the US, historically striving to maintain a state of readiness to respond [23-25]. While infectious respiratory conditions remain a major concern, changing environmental conditions and stresses from expanding industrial, military, and agricultural activities require greater vigilance and laboratory, hospital, and rehabilitation resources. Increasingly prevalent and worsening asthma and COPD, and by extension ACO, could strain the clinical and financial resources of public health services in the US and globally [26]. Better characterization and more accurate diagnosis will help in in the management of ACO, and in the development of better preventive public health strategies to decrease the impact of this clinical entity.

To help to address this gap in knowledge about ACO, this medical record based observational study employed a more rigorous research design—stricter inclusion criteria, plus confirmation of ICD-9 code-based identification of ACO with medical record review—than prior similar claims-based studies. The objective was to estimate the prevalence of ACO in a population of asthma or COPD patients, and describe patterns using an enhanced dual identification approach. Additionally, this study sought to describe medication utilization and health care costs of patients with ACO compared to patients with only asthma or COPD.

## Methods

### Data Source

Data were queried from the HealthCore Integrated Research Database, a single payor health insurance repository of administrative claims data for approximately 43 million members at the time of study. Applicable regulations and the Health Insurance Portability and Accountability Act were followed strictly; the study was approved by the New England Institutional Review Board.

### Study Design and Patient Population

This retrospective cohort study used administrative claims data and medical record reviews between January 1, 2006 and October 31, 2015 (see Multimedia Appendix 1). The index date (first date patients met inclusion criteria) occurred during the intake period, which was between 1 January 2007 and 31 October 2014. Study patients were health plan members, ≥40 years old on index date, and with 12 months pre- and postindex health plan eligibility. Three cohorts were examined (asthma, COPD, or ACO) based on having (1) ≥2 diagnoses (≥30 days apart) for asthma (International Classification of Diseases, Ninth Revision, Clinical Modification [ICD-9-CM] code 493) or COPD (ICD-9 CM codes 491, 492, and 496), (2) ≥2 procedure codes (≥30 days apart) for asthma-related or COPD-related procedures, (3) ≥3 Generic Product Identifier (GPI)-defined prescription fills (≥30 days apart) for asthma or COPD medication, and (4) ≥2 Current Procedural Terminology codes for spirometry tests. Asthma- and COPD-only cohorts had neither diagnostic nor procedure codes for the other disorder. Patients meeting criteria for both asthma and COPD constituted the claims-positive ACO cohort. Patients with a preindex cancer diagnosis were excluded.

### Medical Record Review

ACO was confirmed for the purposes of this study by medical record review of 5000 randomly selected claims-positive patients with ACO during 2015-2016, whose outpatient records were abstracted using a standardized form. COPD was confirmed by persistent airflow obstruction (forced expiratory volume in 1 second [FEV_1_]/forced vital capacity [FVC] less than 0.70) at symptom baseline. Positive computed tomography documentation of emphysema was not required but considered supportive of COPD diagnosis. Medical record confirmation of asthma included any two of the following: allergic rhinitis, chronic sinusitis or eczema, positive skin test or desensitization to environmental allergens, medical history of asthma before age 40 years, or family history of asthma [16]. Smoking status was assessed by medical record review. Patients with medical record features consistent with COPD or asthma as described above we considered to have medical record “confirmed” diagnoses of these disorders, and those with medical record features of both COPD and asthma were considered to have “confirmed ACO” Spirometry criteria for reversibility were not used because reversibility has been shown to occur with COPD as well as asthma, so therefore cannot be used to differentiate COPD from asthma [27].

### Outcome Measures

Demographic variables were measured on the index date. The Deyo-Charlson Comorbidity Index (DCI) [28] score provided a baseline of illness burden. Smoking history was determined from Current Procedural Terminology codes for tobacco cessation counseling (99406, 99407) and use disorder (ICD-9-CM 305.1; V1582). Asthma and COPD medication utilization was assessed with GPI codes. All-cause medical and pharmacy costs were assessed for the 12-month post-index period. Total health care costs included inpatient, emergency department, outpatient, and pharmacy expenditures. Costs were adjusted to 2016 values using the consumer price index for US medical care services [29].

### Statistical Analysis

The study population was characterized with descriptive statistics. Frequencies and percentages were reported for categorical variables; means, medians, and standard deviations for continuous variables. Correspondence analysis was conducted to graphically describe the overlap of asthma and COPD ICD-9 codes with medical records [30-32]. Correspondence analysis is conceptually similar to principal component analysis, but applies to categorical rather than continuous data. In a similar manner to principal component analysis, it provides a means of displaying or summarizing a set of data in two-dimensional graphical form. Comparisons among cohorts for response measures were conducted using paired-comparison *t* tests for continuous variables or *Z* tests for percentage differences for categorical variables. Generalized linear model analyses (GLM) using a log link with gamma distribution were used for cost analyses. The GLM is a flexible generalization of ordinary linear regression that allows for response variables that have error distribution models other than a normal distribution. The GLM generalizes linear regression by allowing the linear model to be related to the response variable via a link function and by allowing the magnitude of the variance of each measurement to be a function of its predicted value. Alpha was set at .05, 2-sided, for statistical significance.

## Results

### Overview

A total of 2,219,034 patients had ≥1 claim for asthma and/or COPD; of those, 20,459 met the inclusion and exclusion criteria and had claims-positive ACO; similarly, 17,156 had claims-positive COPD; and 145,906 had claims-positive asthma (see Multimedia Appendix 1).

### Prevalence

Of the 5000 ACO patients randomly selected for medical record review, 3038 were excluded because of missing records, providers not located, or providers not complying with requests. From the 1962 available records, 1181 were excluded because of absent spirometry results or FEV_1_/FVC values. The remaining 781 successful medical record reviews confirmed ACO in 391 (50.1%) of the patients; 206 (26.4%) with confirmed asthma only; and 106 (13.6%) with confirmed COPD only (see Multimedia Appendices 1 and 2). A total of 78 patients were excluded from analyses as their medical records supported neither an asthma nor COPD diagnosis. We assumed that the proportions of confirmed ACO diagnoses would be similar for patients with medical record reviews compared to study patients overall. Extrapolating the 50.1% ACO confirmation rate to the full claims-positive ACO cohort (20,459 patients) yielded 10,250 patients meeting the confirmation criteria. Dividing this numerator (10,250 patients) by the total number of patients found with ≥1 criterion for asthma or COPD (183,521 patients) resulted in an estimated ACO prevalence of approximately 6%.

### Description of Overlapping Asthma and Chronic Obstructive Pulmonary Disease Diagnoses

Most confirmed ACO patients had several overlapping asthma diagnoses; however, the only overlapping COPD ICD-9 code diagnoses were chronic bronchitis and emphysema (see Multimedia Appendix 3). The most common cross ACO ICD-9 codes were chronic bronchitis mixed with chronic obstructive asthma (51/391, 13.0%), COPD chronic airway disorder occurring with unspecified asthma (50/391, 12.8%), chronic bronchitis comorbid with unspecified asthma (49/391, 12.5%), and patients with both COPD chronic bronchitis and COPD emphysema, as well as chronic obstructive asthma (46/391, 11.8%).

**Figure 1:**
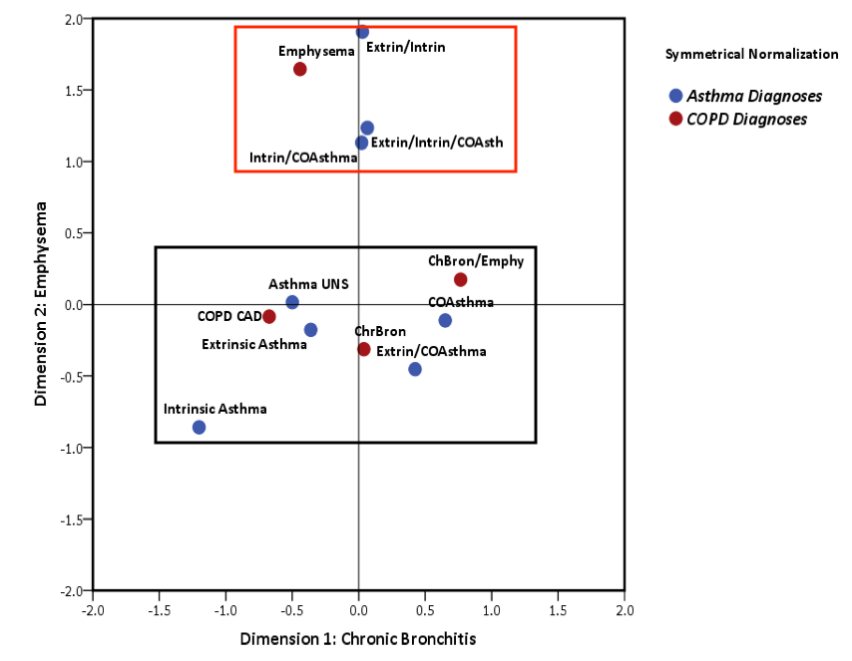
Correspondence analysis biplot of ICD-9-CM subtypes of ACO condition patients. The x- and y-axes are in z-scale metric. Rectangles define two distinct dimensions of patients. The black rectangle encapsulates the historical view that chronic bronchitis is most central to the condition. Note there is variation along x-axis indicating within cluster heterogeneity. The red rectangle captures a distinct dimension that is 1.5 SD distance from the core cluster. This cluster is comprised of multiple mixed asthma diagnoses and emphysema. Overall, there is substantial symptom variation within the ACO condition. Intrin: intrinsic asthma; Extrin: extrinsic asthma; COA: chronic obstructive asthma; Asthma UNS: asthma unspecified; Emphy: COPD emphysema; ChBron: COPD chronic bronchitis; COPD CAD: COPD chronic airway obstruction.

Correspondence analysis confirmed the ACO population. The χ^2^ value was 55.08 (*P*<.001), indicating significant cross-asthma-COPD diagnostic patterns. A 2-dimensional solution accounted for 51.1% of the total variance; dimension 1 for 29.1% and dimension 2 for 22% of total variance (Figure 1). Of the COPD ICD-9 diagnoses codes, chronic bronchitis, chronic airway disease (CAD), and comorbid chronic bronchitis and emphysema diagnoses occurred most frequently; and chronic bronchitis was the most central to the COPD code for the primary cluster of patients. CAD and comorbid chronic bronchitis and or emphysema patterns occurred together less frequently; the codes were about two standard deviations apart. Frequently occurring overlapping asthma symptoms included extrinsic asthma, both unspecified and chronic obstructive forms, also reflecting substantial variation (Figure 1), and heterogeneity in dual diagnosis patterns within ACO. Dimension 2 defined a distinct set of codes comprising multiple mixed asthma diagnoses and emphysema, while differentiating a group of infrequent joint diagnoses with little in common with core ACO characteristics. Few patients (6.1%) were captured in the second cluster.

### Cohort Characteristics and Comorbid Illnesses

A comparison of the mean ages of the claims-based cohorts suggested ACO patients were older (68.4 years, SD 11.4) than asthma patients (53.4 years, SD 9.5) but similar to those with COPD (67.0 years, SD 10.8). In the confirmed cohorts, all groups were of similar age: ACO (68.2 years, SD 11.0), COPD (71.4 years, SD 9.3), and asthma (67.9 years, SD 11.1). Women were the majority in the claims-positive cohorts for both ACO (13,155/20,459, 64.30%) and asthma (99,362/145,906, 68.10%), but not for COPD (8012/17,156, 46.70%). This difference was not observed in the confirmed cohorts with women being the majority in all groups (ACO 232/391, 59.3%; COPD 62/106, 58.5%; and asthma 148/206, 71.8%).

Comorbidity severity (DCI scores) was similar in the claims-positive ACO and COPD cohorts (mean DCI 1.5 for both cohorts, SD 1.7; *P*=.87), but higher than the claims-positive asthma cohort (mean DCI 0.4, SD 0.9; *P*<.001; Tables 1 and 2). In the confirmed diagnosis cohorts, however, comorbidity severity was lower for ACO (mean DCI score 1.3, SD 1.5) versus the COPD cohort (mean DCI score 1.9, SD 2.0; *P*=.007), but similar to the asthma cohort (mean DCI score 1.5, SD 1.6; *P*=.11).

### Smoking History

Smoking was significantly less common in the claims-positive ACO cohort (4133/20,459, 20.2%) versus the COPD cohort (5215/17,156, 30.40%; *P*<.001) but more common than in the claims-positive asthma cohort (6128/145,906, 4.20%; *P*<.001; Tables 1 and 2). No significant difference was seen among the confirmed diagnosis cohorts for claims-assessed smoking: ACO (90/391, 23%) and asthma (35/206, 17.0%; *P*=.27) and COPD (26/106, 24.5%; *P*=.68) cohorts. The difference between the confirmed asthma and confirmed COPD cohorts was statistically significant (*P*=.047). Among the chart reviewed subjects, there was no information on previous or current smoking behavior for 25/206 (12.1%) confirmed asthma cases, 22/106 (20.8%) confirmed COPD cases, and 48/391 (12.3%) confirmed ACO cases. Therefore, the denominators for chart reviewed smoking behavior was 181 for confirmed asthma, 84 for confirmed COPD, and 343 for confirmed ACO. 

**Table 1:** Demographic characteristics and comorbidities for the claims positive cohort.

Demographics		Asthma (n=145,906)	COPD^a^ (n=17,156)	ACO^b^ (n=20,459)	*P* value		
					Asthma vs ACO	COPD vs ACO	Asthma vs COPD
Age (years), mean (SD)		53.4 (9.5)	67.0 (10.8)	68.4 (11.4)	<.001	<.001	<.001
Female, n (%)		99,362 (68.1)	8012 (46.7)	13,155 (64.3)	<.001	<.001	<.001
**Comorbidity**							
	DCI^c^, mean (SD)	0.4 (0.9)	1.5 (1.7)	1.5 (1.7)	<.001	.86	<.001
**Smoking**							
	Claims-assessed, n (%)	6128 (4.2)	5215 (30.4)	4133 (20.2)	<.001	<.001	<.001

^a^COPD: chronic obstructive pulmonary disease.

^b^ACO: asthma-COPD overlap.

^c^DCI: Deyo-Charlson Comorbidity Index.

**Table 2:** Demographic characteristics and comorbidities confirmed diagnosis cohort (based on the random sample of 5000 patients randomly drawn for the claims-positive asthma-chronic obstructive pulmonary disease [COPD] overlap [ACO] cohort).

Demographics		Asthma (n=206)	COPD (n=106)	ACO (n=391)	*P* value		
					Asthma vs ACO	COPD vs ACO	Asthma vs COPD
Age (years), mean (SD)		67.9 (11.1)	71.4 (9.3)	68.2 (11.0)	.69	.003	.003
Female, n (%)		148 (71.8)	62 (58.5)	232 (59.3)	.003	.88	.017
**Comorbidity**							
	DCI^a^, mean (SD)	1.5 (1.6)	1.9 (2.0)	1.3 (1.5)	.11	.007	.10
**Smoking**							
	Claims-assessed, n (%)	35 (17)	26 (25)	90 (23)	.27	.68	.047
	Chart-assessed, n (%)	100 (55.2)^b^	68 (81)^c^	300 (87.5)^d^	<.001	<.001	<.001
	Not documented^e^, n (%)	25 (12.1)	22 (20.8)	48 (12.3)	.67	.001	.001

^a^DCI: Deyo-Charlson Comorbidity Index.

^b^n=181.

^c^n=84.

^d^n=343.

^e^Smoking not documented in medical record.

Thus, the medical record data indicated the confirmed ACO cohort (300/343, 87.5%) had significantly higher percentage of past or present smoking than the confirmed COPD (68/84, 81%; *P*<.001) and confirmed asthma cohorts (100/181, 55.2%; *P*<.001). A significantly greater proportion of patients in the confirmed COPD cohort had a history of smoking, compared with the confirmed asthma cohort (*P*<.001).

### Medication Utilization

Use of asthma and COPD medications was higher among patients in the claims-positive ACO cohort compared with patients in the claims-positive asthma and COPD cohorts (Tables 3 and 4). The only exceptions were the use of long-acting muscarinic antagonists (LAMA), which was higher in the claims-positive COPD (6391/17,156, 37.25%) cohort than in the ACO cohort (6138/20,459, 30.0%; *P*<.001), and long-acting beta2-agonists (LABA) were higher in the COPD cohort (635/17,156, 3.7%) compared to ACO (716/20,459, 3.5%; *P*=.04). Inhaled corticosteroid (ICS) use was not statistically significantly different between the claims-positive asthma and claims-positive ACO cohorts (25,242/145,906; 17.3% vs 3805/20,459; 18.6%; *P*=.09). The claims-positive COPD and ACO cohorts had similar use of short-acting beta-agonist and short-acting muscarinic antagonists (SABA/SAMA; 2728/17,156; 15.9% vs 3110/17,156; 15.2%, respectively; *P*=.76) and SAMA (652/17,156; 3.8% vs 859/20,459; 4.2%, respectively; *P*=.32). In contrast, asthma and COPD medication use was largely similar among patients in the confirmed ACO cohort compared with the confirmed asthma and COPD cohorts (Tables 3 and 4). Compared with the confirmed ACO cohort, the confirmed asthma cohort had lower use of ICS/LABA (59.6% vs 44.7%, respectively; *P*=.001), LAMA (34.0% vs 18.0%, respectively; *P*<.001), SABA/SAMA (18.4% vs 11.2%, respectively; *P*=.02), and LABA (4.6% vs 1.5%, respectively; *P*=.01). Only the use of LAMA was lower in the confirmed ACO cohort compared with the confirmed COPD cohort (34.0% vs 44.3%, respectively; *P*=.05), and only ICS use was higher in the confirmed ACO cohort than in the confirmed COPD cohort (21.2% vs 12.3%, respectively; *P*=.04).

**Table 3:** Chronic obstructive pulmonary disease (COPD) or asthma medication use during the 12-month follow-up period for the claims positive cohort.

Asthma or COPD medication	Asthma (n=145,906), n (%)	COPD (n=17,156), n (%)	ACO^a^ (n=20,459), n (%)	*P* value^b^		
				Asthma vs ACO	COPD vs ACO	Asthma vs COPD
SABA^c^	82,583 (56.6)	7463 (43.5)	12,337 (60.3)	<.001	<.001	<.001
OCS^d^	51,067 (35.0)	7377 (43.3)	11,518 (56.3)	<.001	<.001	<.001
ICS^e^/LABA^f^	49,024 (33.6)	6554 (38.2)	11,191 (54.7)	<.001	<.001	<.001
LTRA^g^	39,103 (26.8)	926 (5.4)	5729 (28.0)	.001	.001	<.001
ICS	25,242 (17.3)	1269 (7.4)	3805 (18.6)	.091	<.001	<.001
SABA/SAMA^h^	3648 (2.5)	2728 (15.9)	3110 (15.2)	<.001	.76	<.001
LABA	3210 (2.2)	635 (3.7)	716 (3.5)	<.001	.04	.01
LAMA^i^	1605 (1.1)	6391 (37.2)	6138 (30.0)	<.001	<.001	<.001
SAMA	1167 (0.8)	652 (3.8)	859 (4.2)	<.001	.32	<.001

^a^ACO: asthma-COPD overlap.

^b^Significance calculated using a *Z* test for differences in column proportions.

^c^SABA: short-acting beta2-agonist.

^d^OCS: oral corticosteroid.

^e^ICS: inhaled corticosteroid.

^f^LABA: long-acting beta2-agonist.

^g^LTRA: leukotriene receptor antagonist.

^h^SAMA: short-acting muscarinic antagonist.

^i^LAMA: long-acting muscarinic antagonist.

**Table 4:** Chronic obstructive pulmonary disease (COPD) or asthma medication use during the 12-month follow-up period for the confirmed diagnosis cohort (based on the random sample of 5000 patients randomly drawn for the claims-positive asthma-COPD overlap [ACO] cohort).

Asthma or COPD medication	Asthma (n=206), n (%)	COPD (n=106), n (%)	ACO (n=391), n (%)	*P* value^a^		
				Asthma vs ACO	COPD vs ACO	Asthma vs COPD
SABA^b^	119 (57.8)	68 (64.2)	249 (63.7)	.16	.93	.28
OCS^c^	112 (54.4)	63 (59.4)	228 (58.3)	.36	.84	.39
ICS^d^/LABA^e^	92 (44.7)	59 (55.7)	233 (59.6)	.001	.47	.07
LTRA^f^	68 (33.0)	23 (21.7)	109 (27.9)	.19	.20	.04
ICS	40 (19.4)	13 (12.3)	83 (21.2)	.60	.04	.11
LAMA^g^	37 (18.0)	47 (44.3)	133 (34.0)	<.001	.05	<.001
SABA/SAMA^h^	23 (11.2)	17 (16.0)	72 (18.4)	.02	.57	.22
SAMA	5 (2.4)	3 (2.8)	21 (5.4)	.09	.28	.98
LABA	3 (1.5)	5 (4.7)	18 (4.6)	.047	1.0	.04

^a^Significance calculated using a *Z* test for differences in column proportions.

^b^SABA: short-acting beta2-agonist.

^c^OCS: oral corticosteroid.

^d^ICS: inhaled corticosteroid.

^e^LABA: long-acting beta2-agonist.

^f^LTRA: leukotriene receptor antagonist.

^g^LAMA: long-acting muscarinic antagonist.

^h^SAMA: short-acting muscarinic antagonist.

**Table 5:** All-cause health care costs during follow-up.

All-cause health care costs			Asthma	COPD^a^	ACO^b^	*P* value^c^
						Asthma vs ACO	COPD vs ACO	Asthma vs COPD
**Claims positive cohort**								
	Patients, n		145,906	17,156	20,459	—	—	—
	**Total costs (US $), mean (SD)**		10,103 (18,987)	25,546 (54,118)	25,307 (42,735)	<.001	.690	<.001
		Inpatient	1836 (13,419)	11,251 (45,205)	10,311 (35,065)	<.001	.003	<.001
		Emergency department	397 (1518)	506 (1707)	701 (2456)	<.001	<.001	<.001
		Outpatient	4682 (9503)	8,826 (21,557)	9050 (18,602)	<.001	.475	<.001
		Prescription	3188 (5079)	4963 (7438)	5594 (8652)	<.001	<.001	<.001
**Confirmed diagnosis cohort**								
	Patients, n		206	106	391	—	—	—
	**Total costs (US $), mean (SD)**		20,311 (23,122)	27,132 (34,680)	19,419 (23,353)	.560	.001	.007
		Inpatient	5973 (16,080)	13537 (28,003)	7026 (18,258)	.497	.030	.011
		Emergency department	587 (1945)	462 (1113)	743 (2548)	.274	.083	.411
		Outpatient	9393 (14,460)	8614 (13,596)	6257 (7020)	.002	.007	.132
		Prescription	4358 (3590)	4518 (3594)	5393 (8579)	.008	.086	.705

^a^COPD: chronic obstructive pulmonary disease.

^b^ACO: asthma-COPD overlap.

^c^Significance calculated using a *Z* test for differences in column proportions.

### Health Care Costs

Mean total health care costs 12 months postindex were significantly higher for patients in the claims-positive ACO cohort compared with the claims-positive asthma cohort (US $25,307 vs US $9966, respectively; *P*<.001), but similar to the claims-positive COPD cohort (US $25,198; *P*=.69; Table 5). Mean costs in the claims-positive ACO cohort were significantly higher than the claims-positive asthma cohort for inpatient (US $10,171 vs US $1811, respectively; *P*<.001), emergency department (US $691 vs US $391, respectively; *P*<.001), outpatient (US $8927 vs US $4618, respectively; *P*<.001), and prescription expenditures (US $8534 vs US $3145, respectively; *P*<.001). Mean costs in the claims-positive ACO cohort were lower than in the claims-positive COPD cohort for inpatient costs (US $10,171 ACO vs US $11,098 COPD; *P*=.003) but higher for emergency department costs (US $692 ACO vs US $499 COPD; *P*<.001).

When mean total costs were compared among the confirmed cohorts, however, the ACO cohort had significantly lower costs than the COPD cohort (US $19,155 vs US $26,762, respectively; *P*=.001) but similar to those of the asthma cohort (US $20,035; *P*=.56). The confirmed ACO cohort had lower mean costs than the confirmed asthma cohort for outpatient (US $6172 ACO vs US $9265 asthma; *P*=.002) and prescription costs (US $5320 ACO vs US $4299 asthma; *P*=.008), and lower mean costs than the confirmed COPD cohort for inpatient (US $6930 ACO vs US $13,353 COPD; *P*=.03) and outpatient costs (US $6172 ACO vs US $8497; *P*=.007).

## Discussion

### Principal Results

The prevalence of ACO in this study population was estimated at 6%, determined by a claims-based definition combined with medical record review to further support the diagnosis. We extrapolated the proportion of medical record confirmed ACO diagnoses from the medical record review (50.1%) to the wider claims-based asthma, COPD, and ACO study population. Medical record review added specificity to ACO diagnoses versus claims alone. Historically in claims-based studies of ACO patients were considered as meeting the definition for ACO if patients had a minimum number of ICD code diagnosis for both COPD and asthma on different occasions. Given the substantial overlap in asthma and COPD symptoms, patients could be diagnosed with either condition by clinicians, this may reflect some degree of ambiguity regarding which clinical diagnosis patients actually are manifesting, therefore reflecting a diagnostic challenge rather than a true clinical overlap syndrome. We have attempted to clarify this situation by going beyond the ICD-9 codes in a sample of patients to identify those patients who meet the traditional claims-based attribution of ACO, but also have corroborating information in the medical record that features of both asthma and COPD actually exist and the ICD-9 codes are to some degree supportable. We were therefore able to define a group of patients who had ICD-9-based characterization of ACO, COPD, and asthma but also ICD-9- and medical record review-based characterization as ACO, COPD, and asthma; and, consequently, compare these two groups. This provided useful information on the condition of ACO but also on the role of claims-based research in the future study of this syndrome.

Current or past tobacco smokers were at higher risk for ACO. Greater proportions of both claims-positive COPD and ACO patients smoked, and the confirmed ACO cohort had a significantly higher percentage of past or present smoking than the confirmed COPD and the confirmed asthma cohorts. Van den Berg and Aalbers suggested two ACO clinical phenotypes: never-, ex-, or current smokers with a history of asthma who have incompletely reversible airflow obstruction; and smokers or ex-smokers with COPD who display increased bronchodilator reversibility [17]. Our data underscored the key association of smoking with ACO as likely contributing to the evolution of the persistent airflow limitation feature of this clinical entity. The difference between confirmed ACO and confirmed asthma was largely the evidence of persistent airflow limitation based on FEV_1_/FVC.

Results from the correspondence analysis question the rationale for including patients with diagnosed emphysema in future studies as they have little in common with the majority of ACO patients. Diagnoses most central to ACO were chronic bronchitis, chronic airway disease, chronic obstructive asthma, asthma not otherwise specified, and extrinsic asthma. Additionally, symptom patterns can present differently. The overlapping diagnostic codes reflecting bronchitis or airway disease may suggest bronchitis symptoms (cough, phlegm production, etc) are more suggestive of ACO versus COPD. This merits further study.

Demographic and comorbidity profiles were similar for confirmed ACO and COPD cohorts. The claims-positive ACO cohort had a greater comorbidity burden than the claims-positive asthma cohort, demonstrating differences between claims-based and medical record-confirmed definitions of ACO. Likewise, evidence in the claims-based cohorts showed greater use of most asthma and COPD medications for ACO patients versus the other two cohorts but results for confirmed cohorts did not show as many significant differences. ICS/LABA, LAMA, SABA/SAMA, and LABA usage was greater in the confirmed ACO versus the confirmed asthma cohort; use of ICSs was greater compared with confirmed COPD cohort, suggesting that ACO might be more responsive to ICSs. The results indicate that ACO patients are prescribed the same amount—or more—asthma and COPD medications as patients with asthma or COPD alone.

Higher ACO versus asthma costs were seen across inpatient, emergency department, outpatient, and pharmacy categories. Costs were lower, however, for all categories for COPD versus ACO patients, except for emergency department services where ACO-attributable costs were significantly greater than COPD-attributable costs. Costs were different in the confirmed cohorts. ACO patients had significatly lower costs than COPD patients, and similar to those in the asthma cohort. Confirmed ACO patients had lower mean costs relative to asthma patients for outpatient and pharmacy services, as well as confirmed COPD patients for inpatient and outpatient services. Possible reasons may include greater treatment responsiveness and less severe disease versus the COPD cohort, although our study was not designed to provide any further clarification.

### Limitations

Despite the strengths inherent in its design, these study results must be viewed against important limitations. Data were from commercially insured patients and results may not be generalizable more broadly. Almost two-thirds (60.1%) of the accessed medical records were excluded primarily because spirometry results or provider FEV_1_/FVC values were missing. Missing data also constrained ACO identification in the claims-positive ACO population.

### Comparisons With Prior Studies

The 6% prevalence estimate of ACO in the study population was lower than in earlier studies, which ranged from 12% to 55% [8,10,12,13,16] but was consistent with the 5.5% estimate from the Majorca Real-Life Investigation in COPD and Asthma study [14], and in line with the 4% to 12% estimates of other recent studies [9,11]. These discrepancies between our study and prior studies might be attributed, in part, to the quantity and quality of available medical records. Only 40% of medical records accessed were complete and usable in confirming the ACO diagnosis and may have provided insufficient information to confirm 49.9% of claims-positive ACO cases. If this was accurate, and assuming that all ACO diagnoses were confirmed by medical record review (100%), under this scenario the estimated ACO prevalence would be 11%, which is consistent with a prior observational study [11].

Our findings of a greater comorbidity burden, medication use, and costs in the claims-positive ACO cohort compared with the claims-positive asthma and COPD cohorts were consistent with prior studies [18]. However, the lower costs observed in the confirmed ACO cohort differed from prior studies that showed ACO patients with higher resource utilization and costs [18,33-35]. This suggests a striking method effect upon results across studies. Costs are critical in public health activities, and they have important implications for all stakeholders. Like the Gerhardsson de Verdier et al claims-based study, which showed costs doubling for ACO versus asthma patients, our study showed an increase (almost 3-fold) for ACO versus asthma alone at 12-months’ follow-up but were similar for ACO and COPD patients in that time frame.

### Conclusions

ACO and asthma patients had similar demographic profiles, and ACO and COPD patients had similar comorbidity burdens. Health care costs for ACO, asthma, and COPD patients were in the same range, but ACO patients received slightly more medication versus asthma or COPD patients. Medical record confirmation of ACO suggested a lower prevalence and other differences than claims-based identification. Such methods-based variations should be considered in future studies.

## Appendix

Multimedia Appendix 1 Study design.

Multimedia Appendix 2 Patients with chart-confirmed asthma and/or chronic obstructive pulmonary disease.

Multimedia Appendix 3 Correspondence table for overlap among ICD-9 subtype diagnoses for medical record confirmed ACO population.

## Abbreviations

ACO: asthma-COPD overlap
CAD: chronic airway disease
COPD: chronic obstructive pulmonary disease
DCI: Deyo-Charlson Comorbidity Index
FEV1: forced expiratory volume in 1 second
FVC: forced vital capacity
GPI: generic product identifier
GLM: generalized linear model
ICD-9: International Classification of Diseases, Ninth Revision
ICD-9-CM: International Classification of Diseases, Ninth Revision, Clinical Modification
ICS: inhaled corticosteroid
LABA: long-acting beta2-agonists
LAMA: long-acting muscarinic antagonists
LTRA: leukotriene receptor antagonist
OCS: oral cotricosteroid
SABA: short-acting beta-agonist
SAMA: short-acting muscarinic antagonist
