# Supplementary figures and images for: Overlap of Asthma and Chronic Obstructive Pulmonary Disease in Patients in the United States: Analysis of Prevalence, Features, and Subtypes
Source: JMIR Public Health Surveill. 2018 Aug 20;4(3):e60. doi: 10.2196/publichealth.9930 (PMC6121140; doi:10.2196/publichealth.9930)

Multimedia Appendix Figure 1. Study Design

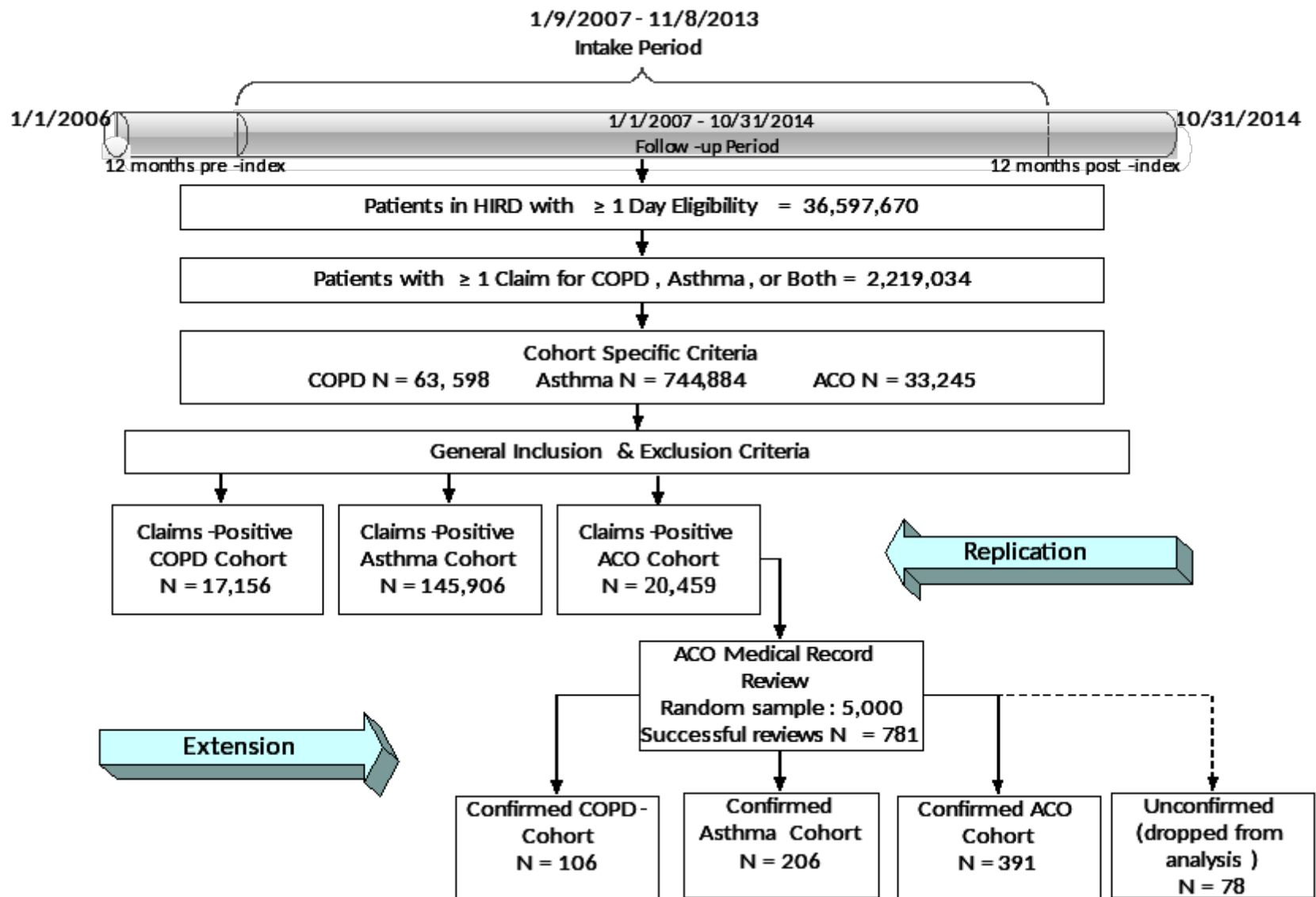

Supplement: Multimedia Appendix 1 [file publichealth_v4i3e60_app1.pdf]
