# Supplementary material for: Overlap of Asthma and Chronic Obstructive Pulmonary Disease in Patients in the United States: Analysis of Prevalence, Features, and Subtypes
Source: JMIR Public Health Surveill. 2018 Aug 20;4(3):e60. doi: 10.2196/publichealth.9930 (PMC6121140; doi:10.2196/publichealth.9930)
Supplement: Multimedia Appendix 2 [file publichealth_v4i3e60_app2.pdf]

Multimedia Appendix Table 1. Patients with chart-confirmed asthma and/or COPD

			**Chart Confirmed Asthma**		**Total**
			**Unconfirmed Asthma**	**Confirmed Asthma**	
Chart Confirmed COPD	Unconfirmed COPD	n (%)	78 (10)	206 (26.4)	284 (36.4)
	Confirmed COPD	n (%)	106 (13.6)	391 (50.1)	497 (63.6)
Total		n (%)	184 (23.6)	597 (76.4)	781 (100)
