# Supplementary material for: Overlap of Asthma and Chronic Obstructive Pulmonary Disease in Patients in the United States: Analysis of Prevalence, Features, and Subtypes
Source: JMIR Public Health Surveill. 2018 Aug 20;4(3):e60. doi: 10.2196/publichealth.9930 (PMC6121140; doi:10.2196/publichealth.9930)
Supplement: Multimedia Appendix 3 [file publichealth_v4i3e60_app3.pdf]

Multimedia Appendix Table 2. Correspondence Table for Overlap among ICD-9 Subtype Diagnoses for Medical Record Confirmed ACO Population

ICD-9 COPD Diagnoses [a]	ICD-9 Asthma Diagnoses[a]								
	Extrinsic / Intrinsic Asthma n (%)	Extrinsic / Intrinsic / Chronic Obstructive Asthma n (%)	Intrinsic Asthma n (%)	Intrinsic / Chronic Obstructive Asthma n (%)	Extrinsic / Chronic Obstructive Asthma n (%)	Extrinsic Asthma n (%)	Chronic Obstructive Asthma n (%)	Asthma Not Otherwise Specified n (%)	Total n (%)
Emphysema n (%)	2 (0.5)	2 (0.5)	0 (0.0)	3 (0.8)	0 (0.0)	4 (1)	4 (1)	9 (2.3)	24 (6.1)
Chronic Bronchitis/ Emphysema n (%)	2 (0.5)	3 (0.8)	0 (0.0)	7 (1.8)	12 (3.1)	8 (2.0)	46 (11.8)	21 (5.4)	99 (25.3)
Chronic Airway Disorder n (%)	1 (0.3)	2 (0.5)	6 (1.5)	6 (1.5)	8 (2.0)	17 (4.3)	19 (4.9)	50 (12.8)	109 (27.9)
Chronic Bronchitis n (%)	4 (1)	3 (0.8)	5 (1.3)	3 (0.8)	16(4.1)	28 (7.2)	51 (13)	49 (12.5)	159 (40.7)
**Total**	9 (2.3)	10 (2.6)	11 (2.8)	19 (4.9)	36 (9.2)	57 (14.6)	120 (30.7)	129 (33)	391

χ^2 = 55.08 (21 degrees of freedom), p = .0001

[a]Asthma and COPD diagnoses observed during pre and post-index periods

Cell percentages are for the medical record confirmed ACOS population total N = 391.

Cells shaded in grey show the primary conditions contributing mass to dimension 1.

Cells shaded in pink show the primary conditions contributing mass to dimension 2.
